# The 2019 *PGDIS* position statement on transfer of mosaic embryos within a context of new information on PGT-A

**DOI:** 10.1186/s12958-020-00616-w

**Published:** 2020-05-29

**Authors:** N. Gleicher, D. F. Albertini, D. H. Barad, H. Homer, D. Modi, M. Murtinger, P. Patrizio, R. Orvieto, S. Takahashi, A. Weghofer, S. Ziebe, N. Noyes, for the International Do No Harm Group in IVF (IDNHG-IVF)

**Affiliations:** 1 Center for Human Reproduction, 21 East 69th Street, New York, N.Y 10021 USA; 2 The Foundation for Reproductive Medicine, New York, N.Y USA; 3 Stem Cell Biology and Molecular Embryology Laboratory, Rockefeller University, New York, N.Y USA; 4 Department of Obstetrics and Gynecology, Medical University of Vienna, 1090 Vienna, Austria; 5 Queensland Fertility Group, Watkins Medical Center, Springhill, Queensland Australia; 6 Molecular and Cellular Biology Laboratory, ICMR – National Institute for Research in Reproductive Health, Mumbai, India; 7 Nextclinic, IVF Zentren Prof. Zech, 6900 Bregenz, Austria; 8 Department of Obstetrics and Gynecology and Reproductive Sciences, Division of Reproductive Endocrinology and Infertility, Yale University, New Haven, CT USA; 9 Department of Obstetrics and Gynecology, Sheba Medical Center, Ramat Gan, Sackler School of Medicine, Tel-Aviv University, Tel-Aviv, Israel; 10 Department of Biomedical Ethics and Department of Obstetrics and Gynecology, The University of Tokyo Graduate School of Medicine, Tokyo, Japan; 11 Ringhospitalet, University Hospital Copenhagen, Copenhagen, Denmark; 12 Donald and Barbara Zucker School of Medicine at Hofstra/Northwell, New York, and Northwell Health’s System, New York, N.Y USA

**Keywords:** Preimplantation genetic screening (PGS), Preimplantation genetic testing for aneuploidy (PGT-A), In vitro fertilization (IVF), Embryo selection, Pregnancy rates, Live birth rates, Miscarriage rates

## Abstract

**Background:**

A recently published Position Statement (PS) by the *Preimplantation Genetics Diagnosis International Society (PGDIS)* regarding utilization of preimplantation genetic testing for aneuploidy (PGT-A) in association with in vitro fertilization (IVF) contained inaccuracies and misrepresentations. Because opinions issued by the *PGDIS* have since 2016 determined worldwide IVF practice, corrections appear of importance.

**Methods:**

The *International Do No Harm Group in IVF (IDNHG-IVF)* is a spontaneously coalesced body of international investigators, concerned with increasing utilization of add-ons to IVF. It is responsible for the presented consensus statement, which as a final document was reached after review of the pertinent literature and again revised after the recent publication of the STAR trial and related commentaries.

**Results:**

*In contrast to the PGDIA-PS*, we recommend restrictions to the increasing, and by IVF centers now often even mandated, utilization of PGT-A in IVF cycles. While PGT-A has been proposed as a tool for achieving enhanced singleton livebirth outcomes through embryo selection, continued false-positive rates and increasing evidence for embryonic self-correction downstream from the testing stage, has led *IDNHG-IVF* to conclude that currently available data are insufficient to impose overreaching recommendations for PGT-A utilization.

**Discussion:**

Here presented consensus offers an alternative to the 2019 *PGDIS* position statement regarding utilization of preimplantation genetic testing for aneuploidy (PGT-A) in association with in vitro fertilization (IVF). Mindful of what appears to offer best outcomes for patients, and in full consideration of patient autonomy, here presented opinion is based on best available evidence, with the goal of improving safety and efficacy of IVF and minimizing wastage of embryos with potential for healthy births.

**Conclusions:**

As the *PGDIS* never suggested restrictions on clinical utilization of PGT-A in IVF, here presented rebuttal represents an act of self-regulation by parts of the IVF community in attempts to control increasing utilization of different unproven recent add-ons to IVF.

## Background

Attributed at least, in part to recently introduced add-ons, live birth rates following fresh non-donor in vitro fertilization (IVF) cycles have substantially declined [1]. This downward trend over the past decade has paralleled a marked increase in the use of preimplantation genetic testing (PGT-A) and of other so-called add-ons to IVF. Unvalidated utilization of add-ons to IVF was first systematically addressed by Harper et al. [2] and has recently, in general, attracted increasing attention in the fertility literature [3] and lay press [4].

As a treatment paradigm in routine IVF, PGT-A mandates cumulative add-ons with their own independent potential to adversely impact IVF outcomes, such as extended blastocyst culture, embryo cryopreservation, frozen embryo transfer and disposal of what the procedure reports as chromosomal-abnormal embryos. PGT-A, therefore, not only, in itself, reduces pregnancy chances as pointed out by Paulson [5], but, secondarily, imposes increased additional interventions with potential negative clinical outcome consequences and financial burden on IVF. PGT-A, therefore, has likely been the most consequential add-on to IVF in the last decade in defining above noted declines in live birth rates all over the world [1].

Based on its own website, the *Preimplantation Genetic Diagnosis International Society (PGDIS)* is a professional society of 262 worldwide members (http://pgdis.org/docs/members2020_0211.pdf), primarily composed of clinicians and laboratory geneticists instrumental in guiding and promoting PGT-A practice. It recently published an updated Position Statement (*PGSIS*-PS) on the subject of PGT-A [6], which sparked the formation of the *International Do No Harm Group in IVF (IDNHG-IVF)* to formulate a response*.* The *IDNHG-IVF* is a consensus-body of clinicians, embryologists and basic scientists, concerned with advocation of insufficiently validated add-ons to IVF. Because of an important recently published study [7] with two accompanying commentaries [8, 9], this communication appears timely.

## Summarizing the argument

The primary objectives of this communication are to voice concerns regarding statements made in the latest *PGDIS-*PS regarding the nonjudicial usage of PGT-A. Here presented conclusions are based on six difficult to refute facts: (i) The hypothesis that PGT-A improves pregnancy and live birth chances in association with IVF and reduces miscarriages, appears no longer sustainable [7, 10]. (ii) That PGT-A does not improve IVF outcomes in good-prognosis patients, suggests that in poorer-prognosis patients PGT-A, likely, adversely impacts outcomes, as first already reported by Mastenbroek et al. over a decade ago [11]. Loss of false-positively diagnosed embryos is more significant in poorer-prognosis patients with small embryo numbers. (iii) Hundreds of chromosomally healthy births following transfer of, by PGT-A reported to be chromosomal-abnormal embryos (“mosaic” and “aneuploid”), have been reported [12], confirming the discarding of embryos with considerable normal pregnancy potential after false-positive PGT-A diagnoses, recently also pointed out by Paulson [5]. (iv) Demonstration that aneuploid embryos have the capacity to self-correct downstream from the blastocyst stage, was first reported in the mouse [13] and, recently confirmed in the human embryonic cell lineage and in human gastruloids [14]. In mice [13] and humans [14], ability to self-correct is significantly lower in extraembryonic trophectoderm than in the embryonic cell lineage of the inner cell mass. Trophectoderm, therefore, for biological reasons alone, cannot reliably represent the inner cell mass. (v) A single trophectoderm biopsy of on average 5–6 cells, as is currently the practice in PGT-A at blastocyst stage, mathematically cannot represent the whole embryo [15]. (vi) In clinical medicine, the responsibility to establish validated evidence in support of a proposed treatment and/or test, rests with proponents of treatments/tests, mandating that such evidence exists before such treatments/tests are integrated into routine clinical practice.

Without further improvements in PGT-A, the *IDNHG-IVF* here suggests that current results obtained with PGT-A should be viewed critically. In opposition to some of the recommendations for laboratory and/or clinical practice proposed by the *PGDIS*-PS, the *IDNHG-IVF*, therefore, advocates limitation on PGT-A usage.

## Addressing the 2019 *PGDIS* position statement (PS)

*PGDIS*-PS 2019 PS [6] is the second statement from this group with the intent of informing PGT-A practice worldwide. A first such document was issued in July of 2016 on the organization’s website (http://pgdis.org/docs/newsletter_071816.html) and by e-mail to membership, establishing PGT-A criteria that have since been followed by most genetic testing laboratories and IVF centers around the world. It is *this* power of the *PGDIS* to influence worldwide PGT-A and IVF practices without even formal publication of documents in a peer-reviewed process, that has created an urgency in rebutting the most recent *PGDIS-*PS which has the potential of steering patients toward add-ons to IVF that may not improve cycle outcomes for many patients and, potentially, even harm some [3].

*PGDIS* statements and activities never suggested any restrictions for PGT-A utilization and, therefore, implicitly have endorsed unrestricted use of PGT-A (and its precursor, preimplantation genetic screening, PGS). *PGDIS*-PS 2019 is in that regard no exception. Devoid of references, it includes the following misleading introductory statement:” *Identification of aneuploid and transfer of euploid embryos has demonstrated improved rates for implantation, pregnancy and live birth per transfer and reduced implantation failures*.”

While mounting reports [7, 10], some succinct commentaries [8, 9] and a restated combined *American Society for Reproductive Medicine* (*ASRM)/Society for Assisted Reproductive Technology (SART)* committee opinion [16], refute such a conclusion, this introductory statement appears clearly meant to convey that PGT-A improves implantation, pregnancy and live birth rates and reduces implantation failure. By using the denominator, “*per transfer,*” the statement is, didactically correct; it, however, at the same time conveys highly misleading information since, as is widely accepted, IVF outcome reporting with reference point embryo transfer excludes poorer-prognosis patients whose embryos may never reach embryo transfer, especially if, as in cases of PGT-A, embryo culture to blastocyst-stage becomes mandatory.

Here is a simple mathematical example for how misleading IVF outcome reporting is with reference point embryo transfer, assuming a clinical trial with a starting patient population of 100 women below age 42 (i.e., relatively young patients): Assume that among those, 15 are excluded from the study because of suboptimal ovarian reserve testing or poor prior response to gonadotropin stimulation. Another 20 are subtracted for lack of embryos reaching the blastocyst stage. In remaining 65 patients, only approximately 1/3 (or approximately 22 women) will have one or more frozen *euploid* blastocyst. Further assuming a 50–60% live-birth rate per single, seemingly euploid blastocyst transferred, at most 12 patients will succeed in having a live birth, *per cycle start*, *−* a live-birth rate of only 12.0%. With reference embryo transfer, the birth rate would, however, be 12/22 (54.6%).

To use such an obviously incorrect statistical outcome assessments as basis for a formal statement in support of outcome benefits from PGT-A is, therefore, inappropriate. Yet, as further discussed below, this practice is unfortunately continuing. Extrapolations of treatment outcomes from best-prognosis to poorer-prognosis patients, has distorted outcome reporting in IVF in innumerable published studies not only related to PGT-A; but it has been almost universally used by proponents of PGT-A in advocating for the procedure.

The previously referred to just-published STAR study offers good examples for correct as well as incorrect outcome reporting [7]. The authors correctly reported results of thawed, elective single-embryo-transfers (eSET) with and without prior PGT-A (the latter relying only on traditional standard manual morphologic embryo assessment). Notably, they found no difference when reporting outcomes *per cycle start* (intent-to-treat). This study received considerable attention in the IVF field because it, quite unequivocally, demonstrated that pregnancy rates did not differ, whether embryos had been tested by PGT-A or not. Furthermore, miscarriage trends, ironically, favored embryos judged by morphologic embryo assessment only.

Inexplicitly, the authors then, however, performed a “post-hoc analysis,” of a subgroup of women between ages 35–40 years, now, suddenly, however, calculating pregnancy rates with reference point *embryo transfer*. As, based on the mathematical example noted above, expected IVF cycle outcomes with PGT-A, now, unsurprisingly improved. Not even really reaching statistical significance (*P* = 0.053), the authors did not hesitate in reporting that, *“…a significant increase in ongoing pregnancy rate”* was observed [7]. Using the authors’ own data, a more recent study described the hubris of this statement in more detail [17].

Schattman in a recent commentary considered the overall study outcome convincing enough to advocate limiting PGT-A to only “*rarest of cases*” and to research studies with appropriate informed consent [9]. In a second commentary, Paulson saw a potentially somewhat brighter future for the procedure if PGT-A could be performed non-invasively from spent media; but he also cautioned against its utilization in women with small embryo numbers (i.e., in poor prognosis patients) [8]. Where and whether PGT-A, ultimately, will find a valid utility, remains at this point still to be seen. Genetic testing in the context of human IVF, now more than ever, must be, however, conscientiously applied and ethically justified, fully acknowledging that the burden of proof for its utilization lies with proponents of the procedure.

## Correcting specific errors in the *PGDIS*-PS

We from here on, follow the section headings of the original document [6].

### Background

What constitutes a *mosaic* embryo, depends on how mosaicism is defined. The *PGDIS*-PS defines mosaicism as, *“presence,’ in a single sample,’ of two or more cell lines with different chromosome sets, which has been observed commonly in a minority of embryos at all stages of preimplantation development”* [6]. The emphasis here is on ‘in a single sample.’ This is, however, not how mosaicism is usually defined: The 2019 *Biology Online Dictionary* defines mosaicism as, “*two or more cell lines anywhere ‘within a complete organism, ‘derived from a single zygote*” (https/:www.biology-online.org/dictionary/Mosaicism). The difference between these two definitions is of great theoretical and clinical importance since inaccurate definitions have led to a host of misrepresentations in respect to PGT-A.

To be “mosaic” a second aneuploid cell line may be anywhere in a given embryo. The *PGDIS*, however, already preceding the 2019 PS, defined an embryo as “mosaic” only if aneuploid cells were detected in a single random, approximately 5–6 cell trophectoderm biopsy. Conventional thinking engendered the notion that only meiotic aneuploidies persist in every cell of an organism. Mitotic aneuploidies, however, are stochastic, and tend to be clonal. Their detection in a single biopsy of TE must, therefore, be considered a random-chance event and mosaicism must be significantly underreported when solely based on single trophectoderm biopsies. Mathematical modeling, indeed, demonstrated that, even under unrealistic best possible circumstances, assuming even distribution of aneuploid cells throughout an embryo, in excess of 25 cells would have to be biopsied to avoid false-positive and/or false-negative results [15]. With clonal distribution, the number of biopsied cells would have to be even larger, clinically an impossible task, if embryos are to survive the biopsy. The *PGDIS* definition of mosaicism is, thus, obviously inaccurate and misleading.

Also grossly misleading is the statement that, “mosaicism” represents an “*intermediate diagnosis somewhere between full aneuploid and normal euploid*.” It offers a mentality of Mendelian genetics, inconsistent with decades worth of human preimplantation embryo investigations. It also fails to fully contextualize the increasingly well-understood importance of chromosomal instability during normal embryo development, including the time period of IVF, and for potentially invasiveness of embryos during implantation [18].

Defined by a *threshold* concept, the *PGDIS* diagnoses mosaicism based on amount of aneuploid DNA, as a percentage of total DNA, in a single trophectoderm biopsy. This concept is not only troubling because of previously noted biological differences between cell lineages in respective abilities to eliminate aneuploid cells (i.e., inner cell mass vs. trophectoderm), but also because advertised cut-offs in aneuploid DNA do not appear based on any validated criteria: Specifically, if the percentage of aneuploid DNA-load in a biopsy specimen is 20–80%, an embryo is considered “mosaic.” Below 20%, a biopsy is considered “normal,” while above 80%, it is deemed “aneuploid” ((http://pgdis.org/docs/newsletter_071816.html). A biological basis for these cut-offs, however, does not exist and appears shortsighted, given the recently developed understanding of human genomic complexities at the very stages of human development, for which PGT-A claims to provide insights.

Moreover, what represents 100% DNA in a single biopsy, is impossible to determine since it depends on the numbers of cells in any given biopsy, which, as any embryologist can attest to, is impossible to determine. It is furthermore impossible to determine how many cells were damaged during biopsy, contributing to fractional loss of DNA content and specimen contamination. Accurate percentages of aneuploid DNA can, therefore, never be calculated. Current definitions of what represents normal, mosaic and aneuploid embryos, must, therefore, also on technical grounds be considered insufficient.

### Overview of new knowledge

From the preceding follows that the *PGDIS*-PS statement, *“mosaic embryos represent 5-10% of all tested embryos*” must also be false. That mosaicism regularly occurs in preimplantation-stage embryos has been known for at least a decade [19]. Unfortunately, claims of analytical precision have, however, not kept up with current claims of specificity and sensitivity. Continuous post-blastocyst-stage self-correction of experimentally induced aneuploid embryos in the mouse added significantly to the conversation about genetic plasticity [13]. Additional information was derived from using bioinformatics of single-cell analyses in human blastocyst-stage embryos. Preliminary data suggest a ca. 50% aneuploidy rate at blastocyst stage (and even higher rates at cleavage stages), with gradual further self-correction downstream [14]. The above-quoted *PGDIS*-PS statement is, therefore, difficult to comprehend. Consequently, given today’s knowledge-base, discarding embryos based on a single TE biopsy appears shortsighted and represents yet another misunderstanding of embryo biology.

### Transfer outcomes from mosaic embryos

Misinterpretations and misstatements also apply to this section of the *PGDIS*-PS. Following the layout of the section, and not necessarily in order of importance, the PS ascribes a “*first published study reporting healthy live births following transfer of apparent mosaic embryos*” to Greco et al. [20]. A first report, preceding Greco et al. by a few weeks, was actually published by other authors [21].

The statement that, *“…. compared to euploid transfers, transfer of mosaic or mosaic segmental embryos do give rise to healthy pregnancies but may be associated with reduced implantation and higher miscarriage rates,*” is unreferenced and in view of recent publications, likely, inaccurate. Unexpectedly high pregnancy/live births of approximately 50% and equally unexpected low miscarriage rates have been reported worldwide following transfers of allegedly chromosomal-abnormal embryos, designated by PGT-A as mosaic or aneuploid-abnormal [12, 20–26]. Moreover, over 400 chromosomal-healthy offspring have so-far been delivered worldwide following such transfers [12]. Whether transfer of (under *PGDIS* – definition) “mosaic” embryos, is at all associated with reduced implantation and/or increased miscarriage rates, therefore, deserves further study and currently available data clearly dispute these propositions.

Another unreferenced claim follows: “… *mosaics with <40% mosaicism had better and mosaics with 40-80% aneuploid mosaics had less likely viable pregnancies*.” Originally, claimed by Munné et.al [24]., this representation was refuted utilizing the study’s own dataset, by establishing ROC curves in 10% aneuploid DNA-increments [27]. This study by Kushnir et al. also further refuted the by the *PGDIS* defined, above noted, mosaicism thresholds. Embryos, designated as “mosaic” should, therefore, be considered transferrable regardless of percentage of aneuploid DNA load. A biopsy with 100% aneuploid DNA, likely, has greater statistical probabilities of being a meiotic aneuploidy than, say, a biopsy with only 20% aneuploid DNA. To argue, however, that 79% aneuploid DNA (“mosaic”) allows transfer, but 81% aneuploid DNA (“aneuploidy”) mandates disposal of embryos, does not reflect biological realities.

### Genetic analysis of mosaic blastocysts

In the Genetic Analysis section of the 2019 *PGDIS*-PS, three studies regarding repeat embryo biopsies are to various degrees misquoted when claiming that repeat biopsies “*have consistently shown a high concordance (95%) of the original aneuploid result with other sites in the embryo, including the ICM and other regions of the TE.”* Gleicher et al. were the first to investigate intra-laboratory and inter-embryo discrepancies in multiple biopsies [21, 22]. They investigated 11 aneuploid embryos (originally diagnosed at a leading reference PGT-A laboratory servicing the whole U.S.), dissected them into 37 anonymized specimens that then, blinded, were sent to a second prominent national laboratory for determinations of concordances or discrepancies in diagnostic results; only 2/11 embryos (18.2%) demonstrated concordance; 4/11 (36.4%) were, on repeat biopsy, normal-euploid, 2/11 (18.2%) were mosaic and 5/11 (45.5%) differed in reported aneuploidies between laboratories. Repeat biopsies of same embryos in the same laboratory differed in 5/10 embryos (50%). Popovich et al. later reported the same 50% non-concordance rate between successive biopsies in same embryos [28]. Such low concordance is, however, exactly what one would expect with sporadic clonal distribution of aneuploid cells in trophectoderm.

Simply based on previously described differences in elimination of aneuploid cells between embryonic (inner cell mass) and extraembryonic cell (trophectoderm) lineages, discrepancies in aneuploidy rates must be expected. Orvieto et al. were the first to report such discrepancies [29]. Popovic et al. reported discrepancies in over one third of tested embryos [28]. When investigating such discordance/concordance, one, of course, can use only single trophectoderm biopsies as reference points since PGT-A relies on such single biopsies. Data reported by Huang et al., therefore, cannot be considered, when he reported a 98.04% concordance because to reach this number, he compared one inner cell mass biopsy to multiple trophectoderm biopsies [30]. Real concordance, therefore, must be significant lower. Multiple biopsies have, indeed, been reported to even demonstrate gender discrepancies [21, 22, 31].

Misrepresentations also affected two manuscripts by Victor et al.: In a first study [25], the authors, indeed, reported better concordance with whole-chromosomes aneuploidy between trophectoderm and inner cell mass than in previously mentioned reports; however, even these authors reported only poor segmental concordance (in 42.9%). With discordance between lineages, concordance between a first and second trophectoderm biopsy, moreover, was only 33.3%. The group’s second manuscript [26], in full concordance with Kushnir et al. [27], demonstrated that amount of aneuploid DNA in mosaic embryos did not correlate with IVF outcomes. An earlier abstract from the same group [32] was also incorrectly referenced since it actually reported very low concordance between multiple trophectoderm biopsies and, regarding increased cell proliferation and cell death, supported previously noted ability for self-correction of embryos.

### How does this affect aneuploidy testing in clinical practice?

Without offering restrictions, the *PGDIS*-PS, thus, continues to support broad adoption of PGT-A in routine IVF. Results of the STAR trial [7] and of accompanying commentaries [8, 9]), suggest, however, otherwise. It now appears established that PGT-A in a number of sequential incarnations, so-far, has failed to establish any validated clinical utility in association with IVF. Schattman advocated that its use be curtailed to clinical trials in efforts to potentially identify subgroups who, after all, may benefit from PGT-A [9]. Paulson sounded a little less restrictive in his commentary [8] but also demanded reconsideration of PGT-A utilization in well-defined patient populations and strongly argued against continuous utilization in older women with small embryo numbers. Interestingly, because Paulson believes that the ineffectiveness of PGT-A is to a large degree caused by damage to embryos during biopsy [5], he also expressed a degree of optimism that non-invasive PGT-A, using cell-free DNA in spent media for diagnosis [33], may be more successful. Some of this optimism may, however, on biological grounds be questioned [34], as recent evidence suggests that chromosomal diversity in early embryo stages likely offers evolutionary advantage. Moreover, if one accepts that embryos downstream continue to self-correct post blastocyst stage, one wonders whether even best diagnostic techniques and technologies can be successful in determining at blastocyst stage which embryo will or will not self-correct. In a quickly arriving era of improving artificial intelligence, one, however, can never rule out surprising advances.

Last in this section, one also must acknowledge two major oversights that, so-far, have plagued the increasingly adversarial dispute surrounding the clinical utilization of PGT-A: One issue completely ignored in the debate, has been the widely acknowledges rule that medical treatments introduced into routine clinical practice should offer validated benefits to patients. As long as such benefits are not established, appropriate informed consent is impossible to obtain and treatments, therefore, are considered experimental. To develop evidence of benefit (i.e., clinical efficacy) is the responsibility of proponents of new treatments. In conjunction with PGT-A, the responsibility to proof efficacy of this diagnostic test, therefore, has been the proponents’; yet, as documented above, the now often even mandated utilization of PGT-A by some IVF centers has come about in absence of such evidence [16], with proponent of the procedure, paradoxically, demanding that opponents of PGT-A offer evidence for lack of efficacy for the procedure..

Secondly, up to this point, discussions in the literature surrounding PGT-A have centered almost exclusively only on whether PGT-A, indeed, offers clinical benefits for IVF outcomes. Whether the procedure adversely affects the IVF process in at least some patients, has, however, been largely ignored [3]. As far back as in 2007, Mastenbroek et al. already pointed out that PGT-A (in those days performed at cleavage stage) in older women actually reduces pregnancy chances [11]. Per Paulson, PGT-A demonstrates a ca. 40% false-positive diagnosis rate [5]. Embryo loss from false positive diagnoses will affect poorer-prognosis patients, of course, more severely than good-prognosis patients who can better afford such losses. With *do no harm* being the first rule of medical practice, ethical considerations regarding PGT-A must, therefore, consider the possibility that, at least selected patient populations, may be adversely affected from being exposed to PGT-A.

### Comments for the laboratory

The concluding remark of the Laboratory Comments section in the 2019 *PGDIS*-PS, was: “*We suggest that the mosaic spectrum be considered a continuous risk gradient ranging from relative lower risk at 20% to higher risk as it approaches 80%. However, clinics should use their own judgment in assigning and the impact this might have on reporting and counseling*.”

Though we here addressed this issue before, it is important to again point out how misleading this statement is in view of published literature [26, 27]. Simply on biological grounds, percentages of aneuploid DNA in a single TEB are unable to reflect an embryo’s overall chromosomal make-up. Consequently, risk grading within what the *PGDIS* considers a “mosaic” trophectoderm biopsy result, may offer a hypothetical mathematical possibility but, because percentages of aneuploid DNA within a trophectoderm biopsy for technical reasons are, basically, impossible to determine, clinical IVF practice does not allow for translation of this threshold concept into distinctions between better and poorer embryos for transfer. Considering that this test is meant to determine which embryos are selected to be transferred or discarded, it is difficult to understand how a test, obviously incapable of fulfilling this challenge, is permitted to be applied in routine clinical practice in determining which embryos are to be disposed of.

### Suggested recommendations to assist in the prioritization of mosaic embryos considered for transfer

The final section of the *PGDIS*-PS, addressing Mosaic Embryo Prioritization, must also be consumed with caution: In offering guidance how to select embryos for transfer, the *PGDIS*-PS refers readers, “*for further guidance to a review by Grati et al.”* In principle, a retrospective analysis of sequential cytogenetic and molecular results of 72,472 chorionic villi samples (and not a review article) [35], as extrapolations of data from prenatal diagnoses to PGT-A has its own problems, the article does not advance prioritizing of allegedly “mosaic” embryos in association with PGT-A,.

## What qualifies an organization to issue practice guidelines?

Reviewing the obvious shortcomings of the 2019 *PGDIS*-PS, also raises the question what qualifies an organization to issue clinical guidelines and how should such guidelines be created. It is not only the opinion of the *IDNHG-IVF* that universal authoritative statements dictating clinical and laboratory practice should originate only after critical and thorough objective data review and, importantly, be devoid of obvious conflicting interests [36]. It is important to consider that, unlike in the past, the world-wide web today serves as a forum for broad distribution of statements, attitudes and dogma – scientific or not – without the benefit of rigorous peer-review prior to publication. The previously referenced July 2016 *PGDIS-*PS, for example, became only electronically available on the society’s website as an unsigned document and without references. Ironically, its’ content now serves to support and encourage the expanding worldwide utilization of PGT-A.

The more recent 2019 *PGDIS*-PS was published by *Reproductive Biomedicine Online* [6], notably, as an unsigned editorial but listing the names of a number of *PGDIS* members who presumably contributed to the manuscript. Our critique of this document should not be understood as a critique of the society, its membership or of the journal that published the document but of the process by which conclusions apparently were reached and included into the 2019 *PGDIS*-PS.

## Summary and conclusions

As increasing discomfort is being expressed with the number of unvalidated add-ons to IVF over the last decade, the primary motivation for this communication has been a growing concern about worldwide exponential increases in PGT-A utilization, likely the single most consequential add-on. Here presented opinions expressed in response to the 2019 *PGDIS*-PS, we hope, will guide and inform future studies of PGT-A, while concomitantly fostering practice restrictions along very recently proposed lines [8, 9]. Conclusions reached are summarized in Table 1. The reasoning for proposed practice changes is clear: unrestricted utilization of PGT-A in absence of outcome improvement for IVF and/or cost-savings as compensatory benefits, is no longer sustainable in the presence of irrefutable evidence that at least some infertility patients undergoing IVF are clinically and financially adversely affected by how PGT-A is currently utilized.

**Table 1:** Key recommendations

• Lacking clear evidence of efficacy in improving PGT-A outcomes and, indeed, raising concerns about causing possible adverse effects on outcomes in at least some patients, the procedure must be considered experimental.	
• Consequently, PGT-A should only be selectively applied within study frameworks and with appropriate informed consent.	
• PGT-A should be considered contraindicated in women with small embryo numbers.	
• Considering existing evidence for high false-positive rates in describing embryos as chromosomal-abnormal by PGT-A, such embryos should no-longer automatically be disposed of but be maintained in cryopreservation, unless disposal is specifically requested by patients.	
• Current knowledge which embryos, by PGT-A designated as chromosomal-abnormal, can be transferred under which outcome expectations, is insufficient, and should be a primary research target.	

Our concerns also extend to the increasing possibility of outside regulatory impositions on IVF, considering recent calls for more of such regulation of IVF add-ons in lay-media [4]. This rebuttal also demonstrated that PGT-A, likely, represents the most consequential add-on introduced to IVF practice over the last decade. Agreement/consensus within our practice community on appropriate interventions into current PGT-A practices emerges as a matter of urgency if IVF is to remain capable of self-regulation in the future.

## Data Availability

N/A
